# Modulation of Cardiac Autonomic Dysfunction in Ischemic Stroke following Ayurveda (Indian System of Medicine) Treatment

**DOI:** 10.1155/2014/634695

**Published:** 2014-05-26

**Authors:** Sriranjini Sitaram Jaideep, Dindagur Nagaraja, Pramod Kumar Pal, D. Sudhakara, Sathyaprabha N. Talakad

**Affiliations:** ^1^Department of Neurophysiology, National Institute of Mental Health & Neurosciences (NIMHANS), Hosur Road, Bangalore 560029, India; ^2^Clinical Research Group, School of Health Sciences, Institute of Transdisciplinary Health Sciences and Technology (FRLHT-ITDHST), Bangalore 560106, India; ^3^Department of Neurology, National Institute of Mental Health & Neurosciences (NIMHANS), Hosur Road, Bangalore 560029, India; ^4^Advanced Center for Ayurveda in Mental Health and Neurosciences, National Institute of Mental Health & Neurosciences (NIMHANS), Hosur Road, Bangalore 560029, India

## Abstract

*Objectives*. Cardiac autonomic dysfunction in stroke has implications on morbidity and mortality. Ayurveda (Indian system of medicine) describes stroke as *pakshaghata*. We intended to study the effect of Ayurveda therapies on the cardiac autonomic dysfunction. *Methods*. Fifty patients of ischemic stroke (middle cerebral artery territory) (mean age 39.26 ± 9.88 years; male 43, female 7) were recruited within one month of ictus. All patients received standard allopathic medications as advised by neurologist. In addition, patients were randomized to receive physiotherapy (Group I) or Ayurveda treatment (Group II) for 14 days. Continuous electrocardiogram and finger arterial pressure were recorded for 15 min before and after treatments and analyzed offline to obtain heart rate and blood pressure variability and baroreflex sensitivity (BRS). Results were analysed by RMANOVA. *Results*. Patients in Group II showed statistically significant improvement in cardiac autonomic parameters. The standard deviation of normal to normal intervals,and total and low frequency powers were significantly enhanced (*F* = 8.16, *P* = 0.007, *F* = 9.73, *P* = 0.004, *F* = 13.51, and *P* = 0.001, resp.). The BRS too increased following the treatment period (*F* = 10.129, *P* = 0.004). *Conclusions*. The current study is the first to report a positive modulation of cardiac autonomic activity after adjuvant Ayurveda treatment in ischemic stroke. Further long term studies are warranted.

## 1. Introduction

Stroke leads to dysfunction of the autonomic nervous system, including cardiovascular, gastrointestinal, genitourinary, thermoregulatory, and sudomotor systems [1]. The cardiovascular regulation is affected due to aberrant activity in the central areas regulating these functions or their pathways [2]. This has been associated with fatal complications [3]. Heart rate variability (HRV), blood pressure variability (BPV), and baroreflex sensitivity (BRS) have been identified as subtle markers of such cardiac dysregulation, persisting in the chronic state and associated with poor prognosis [4–8]. Effective strategies to modulate cardiac autonomic dysfunction may hence provide better prognosis and survival. The possibility of enhancing the baroreflex as a therapeutic strategy in acute stroke has been proposed [9]. Though *β* blockers can influence baroreflex by vagal modulation [10], they are associated with potential harm [11, 12].

From an Ayurveda perspective, stroke is recognized as* pakshaghata* (hemiplegia), a disease attributed to an aberration of* vatadosha* (a physiologic entity). Cardiac dysregulation is understood as a secondary consequence of this process [13]. The diverse treatments advocated in Ayurveda for this disease primarily harmonize the aberrant physiology. Hence, we hypothesized that the Ayurveda therapy may also modulate the cardiac dysregulation. The current study aimed to evaluate the effect of adjuvant Ayurveda therapies on cardiac dysregulation in ischemic stroke using HRV, BPV, and BRS parameters.

## 2. Patients and Methods

The present study was conducted in the Departments of Neurophysiology, Neurology and Advanced Center for Ayurvedic Research in Mental health and Neurosciences, National Institute of Mental Health and Neuro Sciences (NIMHANS), Bangalore. The study was a comparative randomized clinical evaluation, approved by the Institute Ethics Committee, Advanced Center for Ayurvedic Research in Mental health and Neurosciences, NIMHANS. Patients and/or relatives were explained about the nature and design of the study and informed consent was obtained.

### 2.1. Inclusion and Exclusion Criteria

Patients diagnosed with ischemic stroke by a neurologist and as* pakshaghata* (hemiplegia) by an Ayurveda physician by thorough clinical examination and imaging were screened from casualty section, stroke unit, and neurorehabilitation section, NIMHANS. Patients were included if aged between 20 and 60 years, of either gender; with first time ischemic stroke in middle cerebral artery (MCA) territory diagnosed by history, clinical examination and CT scan/MRI of brain, between 10 and 30 days of stroke, with stable neurological status following the necessary acute phase treatment after stroke, motor weakness of at least one limb of MRC Grade 0–3 and fulfilling the criteria to undergo Ayurveda therapies. Patients were excluded if sensorium was altered or severely aphasic to comprehend simple instructions, associated with comorbidities such as uncontrolled hypertension, diabetes, or other systemic diseases, pregnant or lactating. All patients were admitted in the Advanced Ayurvedic Research Institute for Mental Health and Neurosciences, NIMHANS, and randomized to receive the following for a period of 15 days.

### 2.2. Intervention

Group I received conventional treatment including antiplatelet agents or anticoagulants, antihypertensives, and medications for diabetes mellitus hyperlipidemia along with physiotherapy and speech therapy as required. Group II received conventional treatment and speech therapy as in Group I. In addition they received adjuvant Ayurveda treatment consisting of (a) external therapies such as* Abhyanga* (methodical massage) with* Niramisha mahamasha taila* and* Bhashpa svedana* (steam therapy) from day 1 to day 14,* Matra basti* with 60 mL of* Balaswagandhadi taila* from day 8–14 and (b) internal medication of* Ashtavarga kashaya* orally, 15 mL TID with 15 mL warm water and* Ksheerabala 101* orally, 5 drops BD with 15 mL warm water. Refer Table 3 for details of medicine used.

### 2.3. Assessment Criteria

Patients were evaluated with cardiac autonomic function tests which included heart rate variability (HRV), blood pressure variability (BPV), and baroreflex sensitivity (BRS), before and after the treatment. The resting heart rate (HR) and blood pressure (BP) recordings were carried out in the autonomic laboratory, Department of Neurophysiology, NIMHANS under standardized conditions [14, 15]. Patients were advised to abstain from alcohol and nicotine for 24 hours before evaluation. The tests were performed in a silent room maintained at a temperature of 22–26°C, between 8 am and 11 am. Patients were advised to have light breakfast two hours prior to the tests and empty bowel and bladder before the tests. They were familiarized with the laboratory settings and briefed about the tests. Recordings were done after 30 minutes of supine rest.

#### 2.3.1. Resting HRV

Lead II electrocardiogram (ECG) and breathing signals were conveyed through analog digital converter (Power lab, 16 channels data acquisition system, AD Instruments, Australia) with a sampling rate of 1024 Hz. MLS 310 Module was used to analyze the different HRV measures. It was ensured that patients were breathing at normal respiratory rate of 12–15 breaths/min by recording the respiratory movements using stethograph. The data were stored in PC and analyzed offline using an automatic programme that allowed visual checking of the raw ECG and breathing signals. Fifteen-minute basal recordings were stored and later, a 5-minute artifact/ectopic free segment was analyzed to obtain time domain and frequency domain parameters of HRV as per the guidelines of task force report [16].

#### 2.3.2. Resting BPV and Spontaneous BRS

Blood pressure was recorded using the Finometer (Finapres Medical Systems (FMS), The Netherlands). Following height correction, physiological and return to flow calibrations, the finger arterial pressure was recorded continuously for 15 min. The Physical was then turned off during the recording. The recorded data was downloaded offline using Finolink software provided by FMS and stored in a PC. All recordings were scanned and 5-minute artifact/ectopic free segment was analyzed using Nevrokard cardiovascular parameter analysis (CVPA) software (version 2.1.0) to obtain the time domain and frequency domain parameters of blood pressure variability. Baroreflex sensitivity (BRS) values were derived by sequence and spectral methods using the same software as per guidelines [17, 18].

### 2.4. Control Data

To assess the presence and extent of cardiac autonomic dysfunction in the ischemic stroke patients, similar ECG data was obtained from 30 clinically healthy subjects.

### 2.5. Description of Ayurveda Therapies

#### 2.5.1. *Abhyanga* (Therapeutic Massage)

*Abhyanga* was performed during morning hours (8 am–12 noon). Patient was advised to empty bowel and bladder before starting the therapy. Patient was made to lie in supine posture over the* Droni* (*Abhyanga* table). Requisite quantity of* Niramisha Mahamasha taila* (medicated oil) was warmed on a water bath and applied on the patient's body. The entire body was then massaged by 2 masseurs with uniform pressure using long strokes and in circular motion, as required [19].

#### 2.5.2. *Svedana* (Sudation)

*Bhashpa sveda* variety of* Svedana* was done. The patient was made to sit in a* Bhashpa svedana yantra* (customized wooden box) into which warm vapors were passed. This induced perspiration in the patient. This was continued for 10–15 min depending on the patients' tolerance level [19].

#### 2.5.3. *Basti* (Therapeutic Enema)

*Matra basti* (therapeutic oil enema) variety of* basti* was used. The procedure was performed after noon (between 12 noon and 3 pm). Patients were advised to take light food and empty bowel and bladder before starting the therapy. Local* Abhyanga* and* svedana* was performed around the gluteal region. Following this, the patient was advised to lie down in the left lateral position on* Abhyanga* table with left led stretched out and the right leg flexed at the knee and hip joints. The patient's left arm was placed beneath his head. A pinch of* Saindhava lavana* (black salt) was mixed in 60 mL of lukewarm* Balaswagandhadi taila* and drawn into a syringe. A rubber catheter was attached to enema syringe and its tip was lubricated with the same oil. The catheter was then introduced into the anus (up to 4 inches length). Patient was advised to breathe slowly and deeply and the oil was steadily pushed into the rectum. The patient was then made to lie supinely while his legs were raised at the hips for 3-4 times and the gluteal region was massaged gently for few seconds. Patient was advised to retain the enema as long as possible [19].

### 2.6. Statistical Methods

Statistical analysis was performed using SPSS. Chi-square and Fisher Exact test was used to find the significance of categorical data comparison between two groups of patients. Independent sample “*t*” test was carried out to compare (a) the HRV variables between pakshaghata patients and healthy subjects and (b) the baseline characteristics of the two groups of patients. 2 × 2 repeated measures analysis of variance (RMANOVA) was used to find the significance of study parameters within and between the two groups of patients. The two independent factors included in the analysis were (1) treatment (between-subjects factor) with two levels: conventional treatment with physiotherapy and conventional treatment with Ayurveda and (2) time (within-subjects factor) with two levels: baseline and after 2 weeks. The main effects when found to be significant were further tested for the direction of differences by using pairwise comparisons adjusted by Bonferroni's method. Values are represented as mean ± SEM and significance was considered if *P* < 0.05.

## 3. Results

### 3.1. Demographic Details

Fifty patients satisfying the study criteria were recruited and allotted to receive treatment. Baseline evaluation of the demographic details of patients indicated the homogeneity in age, gender, height, weight, body mass index, risk factors predisposing to stroke, duration of illness, and side of infarct in both the groups (Table 1). Two patients in Group II were unable to stay in the hospital for the entire duration of treatment and hence dropped from the study. HRV could not be analyzed in 12 patients (Group I-8, Group II-4) due to the presence of artifacts in the ECG. BPV and BRS analysis was done in a subsample of 24 cases (Group I-11, Group II-13).

### 3.2. Comparison of Ischemic Stroke Patients with Healthy Controls

At baseline, the stroke patients demonstrated significantly lower mean NN and higher heart rate when compared with healthy controls. In addition, the time domain measures SDNN and RMSSD and frequency domain measures TP, LF, HF, and HFnu were significantly reduced and the LF/HF ratio was significantly higher (Table 2).

### 3.3. Effect of Treatments on HRV Measures

**Figure 1:**
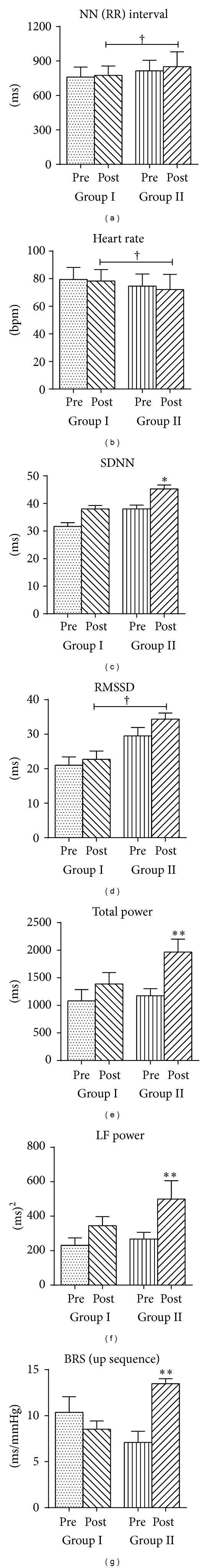
Effect of intervention on heart rate variability measures and baroreflex sensitivity (a) NN—R-R interval (ms); (b) HR—heart rate (bpm—beats per minute); (c) SDNN—standard deviation of NN intervals (ms); (d) RMSSD—square root of the mean of the sum of squares of differences between adjacent NN intervals (ms); (e) TP—total power (ms^2^); (f) LF—low frequency power (ms^2^); (g) BRS—baroreflex sensitivity (ms/mm Hg); values expressed as Mean±SEM; *P < 0.05, **P < 0.01, pre versus post, within group comparison; ^†^ *P* < 0.05 Group I versus Group II at 2 weeks.

Significant treatment effect over time was observed for SDNN (*F* = 8.16, *P* = 0.007), TP (*F* = 9.73, *P* = 0.004), and LF power (*F* = 13.51, *P* = 0.001). Adjusted pairwise comparisons for time showed improvement in these parameters in the group treated with adjuvant Ayurveda therapies. There was also a significant group effect for mean NN (*F* = 6.41, *P* = 0.016), HR (*F* = 5.88, *P* = 0.021), and RMSSD (*F* = 5.87, *P* = 0.021) and adjusted pairwise comparison for group was significant for RMSSD in the group treated with adjuvant Ayurveda therapies at 2 weeks (*P* = 0.025). The LF/HF ratio showed marginal group effect (*F* = 3.34, *P* = 0.076). Pairwise adjustment for group comparisons indicated marginally lesser LF/HF ratio (*P* = 0.067) in the patients treated with adjuvant Ayurveda therapies at 2 weeks. Though there was increase in LF power, no significant change in LFnu or LF/HF ratio in the group treated with adjuvant Ayurveda therapies (Figures 1(a)–1(f)).

### 3.4. Effect of Treatments on BPV and BRS Measures

The SBP, DBP, and MBP showed a trend for decrease in both the groups but it was not statistically significant. We also did not find any significant changes in the time domain and frequency domain measures of BPV following treatment in both the groups (data not shown). Among the sequence and spectral parameters of spontaneous baroreflex, the Up BRS was significantly enhanced in patients treated with adjuvant Ayurveda therapies after pairwise adjustment for time and group at 2 weeks (Figure 1(g)).

## 4. Discussion

The science of Ayurveda has evolved by in-depth observation and experience of scholars. However, the potential benefits of this science need to be evaluated with objectivity. The current study demonstrated that cardiac autonomic functions can be used as a marker to detect one facet of aberrant physiology in* pakshaghata* (hemiplegia) and that a whole system Ayurveda treatment protocol as used here was able to modulate the same.

To the best of our knowledge, this is the first study to evaluate the effect of adjuvant Ayurveda therapies on cardiac autonomic dysfunction in ischemic stroke. The study has used a whole system treatment protocol, adapted from Ayurveda classics keeping in mind convenience for clinical application. This is a positive deviation from single drug clinical trial modules that may be ineffective to capture the essence of Ayurveda treatments [20]. However, we were limited by factors including short follow-up period that prevented evaluation of lasting effect of the treatment. Also, HRV could not be analyzed in few patients (*n* = 12) due to the presence of artifacts. BPV and BRS analysis was done in a subsample of 24 cases. However, compared to the total sample, the subsample analysis was not significantly different indicating that subsample is representative of the total sample.

In the current study, when compared with healthy controls, the patients with ischemic stroke demonstrated significantly lower mean NN and higher heart rate, time domain measures (SDNN, RMSSD) and frequency domain measures (TP, LF, HF, and HFnu) were reduced, and the LF/HF ratio was significantly higher indicating definite cardiac autonomic dysfunction. These findings of altered autonomic activity with increased sympathetic activation correlate with earlier studies on ischemic stroke [6, 14, 21, 22]. The aberrant neurocardiac regulation persists even in the postacute phase [23] and abnormal heart rate dynamics has also been associated with poststroke mortality [24].

Following intervention, it was observed that the patients receiving adjuvant Ayurveda therapies demonstrated significant enhancement of time domain (SDNN) and frequency domain parameters (TP, LF power) of HRV. There was also a significant group effect for mean NN and RMSSD and adjusted pairwise comparison for group was significant in the group treated with adjuvant Ayurveda therapies. This combined with a trend for increase of the HF power and a marginally lesser LF/HF in this group. Though there was increase in LF power, no significant change was observed in LFnu or LF/HF ratio. Among the BPV measures, the SBP, DBP, and MBP showed a trend for decrease in both the groups but it was not statistically significant. We also did not find any significant changes in the other BPV parameters following treatment in both the groups. Among the sequence and spectral parameters of spontaneous baroreflex, the Up BRS was significantly enhanced and the *α*HF also showed a trend for increase following adjuvant Ayurveda therapies.

The time domain measure SDNN and frequency domain measure of TP are robust indicators of overall variability and reflect vagal activation. RMSSD too mirrors the parasympathetic influence on the HRV [16, 25]. The physiological implications of LF power have been conflicting. Earlier studies have demonstrated LF power to be an indicator of sympathetic tone largely [16, 26, 27] while a parasympathetic contribution is also hypothesized [28]. Recent work in this area, however, suggests that the LF component of HRV reflects the baroreflex gain and hence interventions that increase the LF power may modulate the baroreflexes in turn affecting the cardiac autonomic outflow [29–31]. It is also debated that reduced LF power can exist despite clinically evident sympathetic overactivity and this can be attributed to a reduced baroreflex modulation amongst others [32]. In our study too, it was observed that the LF power of the ischemic stroke patients was significantly lower than the healthy controls at baseline, indicating an initially reduced baroreflex function. However, the subsequent increase in most of the HRV components compounded by the increased BRS following adjuvant Ayurveda therapies indicates a positive cardiac autonomic regulation amongst others.

In the present study, we used a whole system approach combining different modes of treatment as mentioned in Ayurveda. These treatments are indicated for neurological illnesses generally and* pakshaghata* (hemiplegia) specifically. Most of the herbs used as internal medication in the current study have been studied for their antioxidant and neuroprotective activity including* Bala* (*Sida cordifolia*) in* Ksheerabala 101* [33, 34],* Devadaru* (*Cedrus deodara*) [35],* Lashuna* (*Allium cepa*) [36, 37],* Shunti* (Zingiber officinale) [38], and so forth. The release of free radicals in the brain is well established in acute and chronic stages of ischemic stroke [39, 40] and antioxidant therapy is seen to be beneficial in late treatment of ischemic stroke too [41]. The therapeutic procedures included* Abhyanga* (methodical massage),* Svedana* (sudation), and* Basti* (colonic administration. Different massage techniques are known to enhance vagal activity [42, 43]. A study on oriental massage demonstrated an improved profile of cardiovascular autonomic regulation [44]. The benefits of* svedana* (sudation) may be akin to that of thermal therapy in the saunas. It is observed that repeated sauna improves cardiac function and hemodynamics [45]. Parasympathetic activity is enhanced and sympathetic activity also reduced on HRV analysis following application of heat and steam generating (HSG) sheets [46, 47].

A probable mode of action of the therapies involved has been put forth by way of evidence that we could gather from contemporary scientific observations. Further critical evaluation of the individual components and whole medicines is required for more definitive claims.

## 5. Conclusions

*Acharya Sushruta says* “it is allowed to interpret concepts of a discipline through premises of other sciences to comprehend wider connotations with an ultimate objective of common good” [52]. The present study was an endeavor in this direction. There is evidence from the current data and literature to support the usefulness of adjuvant Ayurveda therapies in modulating cardiac autonomic dysfunction in ischemic stroke. However, further studies that can incorporate more aspects of individualized whole system protocols of Ayurveda treatment and in a larger population are definitely warranted to enhance the benefits of the science.

## Figures and Tables

**Table 1:** Demographic details of the patients.

Variable	Group I (*n* = 25)	Group II (*n* = 25)	*P* value
Age (in years)	40.12 ± 9.31	38.40 ± 10.50	0.543
Gender (M : F)	20 : 5	23 : 2	0.111
Height (cm)	168.08 ± 2.04	170.84 ± 1.43	0.274
Weight (kg)	66.72 ± 2.38	67.01 ± 2.51	0.936
BMI (kg/m^2^)	24.04 ± 0.78	23.33 ± 0.76	0.519
Duration of illness (days)	14.96 ± 4.16	17.20 ± 5.74	0.121
Smoking	14	17	0.718
Alcohol	15	17	0.860
Hypertension	2	3	1.000
Diabetics	2	2	1.000
Right side infarct	10	8	—
Left side infarct	15	17	—

M: male; F: female; BMI: body mass index; values expressed as mean ± SD.

**Table 2:** Comparison of heart rate variability (HRV) measures between healthy controls and ischemic stroke patients.

HRV variables	Controls (*n* = 30)	Patients (*n* = 38)	*P* value
NN interval (ms)	869.96 ± 23.15	795.67 ± 16.22	0.009**
HR (bpm)	70.44 ± 1.88	76.08 ± 1.61	0.026*
SDNN (ms)	48.01 ± 3.42	36.47 ± 2.25	0.004**
RMSSD (ms)	38.06 ± 4.13	28.00 ± 2.80	0.027*
TP (ms^2^)	2489.00 ± 337.06	1286.63 ± 148.50	0.000***
LF (ms^2^)	527.16 ± 62.53	290.11 ± 34.87	0.001**
LFnu	44.92 ± 2.71	49.01 ± 3.13	0.481
HF (ms^2^)	812.62 ± 196.77	364.71 ± 90.81	0.005**
HFnu	49.18 ± 2.61	41.87 ± 3.30	0.042*
Sympathovagal balance	1.09 ± 0.13	1.66 ± 0.20	0.043*

NN: R-R interval (ms); HR: heart rate (bpm: beats per minute); SDNN: standard deviation of NN intervals (ms); RMSSD: square root of the mean of the sum of squares of differences between adjacent NN intervals (ms); TP: total power (ms^2^); LF: low frequency power (ms^2^); HF: high frequency power (ms^2^); nu: normalized units; SVB: sympathovagal balance; values expressed as mean ± SEM; *P < 0.05, **P < 0.01, ***P < 0.001.

**Table 3:** Medicines used.

Ingredients	
Sanskrit name	Latin name
(1) *Ashtavarga kashaya* [48]	
Bala	*Sida cordifolia *
Sahachara	*Barleria prionitis *
Eranda	*Ricinus communis *
Shunti	*Zingiber officinale *
Rasna	*Vanda roxburghii *
Suradruma	*Cedrus deodara *
Sinduvara	*Vitex negundo *
Lasuna	*Allium cepa *

(2) *Ksheerabala 101* [49]	
Balamoola	*Sida cordifolia *
Tila taila	Gingelly oil
Goksheera	Milk
Balamoola kalka	*Sida cordifolia *

(3) *Balaswagandhadi thailam* [50]	
Ashwagandha	*Withania somnifera *
Bala	*Sida cordifolia *
Tila taila	Gingelly oil
Lodhra	*Symplocos racemosa *
Sarjikakshara	
Bidara	*Zizyphus jujuba *
Haridra	*Curcuma longa *
Daruharidra	*Berberis aristata *
Renuka seeds	*Vitex negundo *
Kushta	*Saussurea lappa *
Musta	*Cyperus rotundus *
Srigandha	*Santalum album *
Sariva	*Hemidesmus indicus *
Katukarohini	*Piccrorhiza kurro *
Shatahva	*Anethum sowa *
Devadaru	*Cedrus deodara *
Manjistha	*Rubia cordifolia *
Yashti	*Glycyrrhiza glabra *
Usheera	*Vetiveria zizanioides *
Ksheera	Milk

(4) *Niramisha mahamasha taila* [51]	
Masha	*Phaseolus mungo *
Bilva	*Aegle marmelos *
Agnimantha	*Clerodendron latifolium *
Shyonaka	*Oroxylum indicum *
Kashmiri	*Gmelina arborea *
Patala	*Stereospermum suaveolens *
Brihati	*Solanum xanthocarpum *
Kantakari	*Solanum indicum *
Gokshura	*Tribulus terrestris *
Salaprni	*Desmodium gangeticum *
Prishnaparni	*Uraria picta *
Jala	Water
Tila taila	Gingelly oil
Ksheera	Milk
Ashwagandha	*Withania somnifera *
Vidari	*Ipomea digitata *
Shatavari	*Asparagus racemosus *
Kachora	*Hedychium spicatum *
Devadaru	*Cedrus deodara *
Bala	*Sida cordifolia *
Rasna	*Vanda roxburghii *
Prasarini	*Paederia foetida *
Kushta	*Saussurea lappa *
Shatahva	*Anethum sowa *
Parushaka	*Grewia asiatica *
Bharangi	*Clerodendrum serratum *
Punarnava	*Boerhavia diffusa *
Pippali Mula	*Piper longum *
Chitrak	*Plumbago zeylanica *
Jivanti	*Leptadenia reticulata *
Sariva	*Hemidesmus indicus *
Mashaparni	*Teramnus labialis *
Mudgaparni	*Phaseolus trilobus *
Guduchi	*Tinospora cordifolia *
Yashti	*Glycyrrhiza glabra *
Saindhava lavana	Rock salt
Hingu	*Ferula foetida *
